# Analytical Life Cycle Assessment as a 10-Stage Framework for Evaluating and Improving the Sustainability of Analytical Workflows

**DOI:** 10.1021/acs.analchem.6c03658

**Published:** 2026-08-24

**Authors:** Fotouh R. Mansour, Marcello Locatelli, Khalid M. Omer, Sameera Sh. Mohammed Ameen, Alaa Bedair

**Affiliations:** † Pharmaceutical Analytical Chemistry Department, Faculty of Pharmacy, Tanta University, Tanta 31111, Egypt; ‡ Department of Medicinal Chemistry, Faculty of Pharmacy, King Salman International University (KSIU), Ras Sidr, South Sinai 46612, Egypt; § Department of Science, University “G. d’Annunzio” of Chieti-Pescara, Via dei Vestini 31, Chieti 66100, Italy; ∥ Department of Chemistry, College of Science, University of Sulaimani, Qliasan Street, Sulaymaniyah, Kurdistan region 46002, Iraq; ⊥ Department of Chemistry, College of Science, 557749 University of Zakho, Zakho, Kurdistan Region 42002, Iraq; # Department of Analytical Chemistry, Faculty of Pharmacy, 392053 University of Sadat City, Sadat City 32958, Egypt

## Abstract

Analytical life cycle assessment (ALCA) extends traditional life cycle assessment (LCA) methodology to the unique context of analytical chemistry, clinical diagnostics, environmental monitoring, and forensic testing. Unlike conventional LCA, which focuses on physical products with linear supply chains, ALCA addresses the distinctive environmental, operational, and economic characteristics of analytical workflows. This tool presents a comprehensive 10-stage ALCA framework consisting of sampling, transportation, storage, pretreatment, sample preparation, premeasurement, measurement, waste disposal, assessment, and reporting/decision making. Each stage is defined, its types are described, and its potential impact on the overall analytical process is analyzed. A dual-threshold assessment rubric is introduced, combining absolute benchmarks (green, yellow, and red zones based on best practices) with relative hotspot identification (comparison to the average contribution per stage). Five hypothetical case studies demonstrate the application of ALCA to assess energy consumption, water consumption, carbon footprint, active analyst time, and cost. The case studies show how the dual rubric identifies critical hotspots, guides improvement decisions, and prevents the problem of all stages appearing green, while the total impact remains unacceptably high. ALCA provides a practical, actionable framework for laboratories seeking to balance analytical reliability, practicality, and environmental sustainability in accordance with the white analytical chemistry principle. The tool is freely available for end-users at bit.ly/ALCA2026.

## Graphical abstract

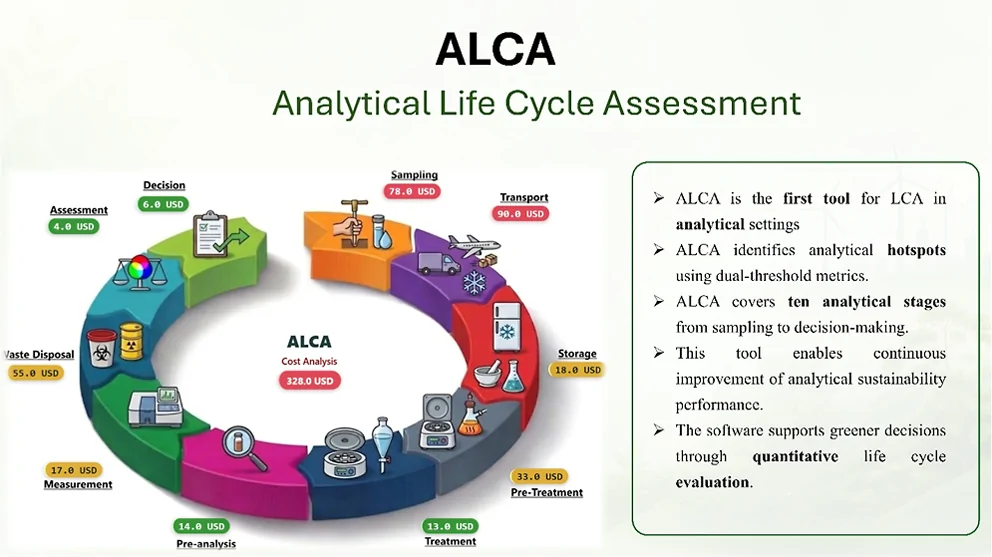

## Introduction

Life cycle assessment (LCA) has long been established as a robust, standardized methodology for evaluating the environmental burdens associated with a product, process, or service across its entire lifespan, from raw material extraction through production, use, and final disposal.^1^ By quantifying energy and material inputs^2^ as well as emissions to air, water, and land,^3^ traditional LCA helps industries,^4^ policymakers, and researchers identify environmental hotspots, compare alternative systems, and avoid problem-shifting from one life cycle stage to another.^5^ Its importance is undeniable in sectors such as manufacturing,^6^ construction,^7^ packaging,^8^ and energy,^9^ where physical goods and large-scale processes dominate the environmental footprint. However, a critical gap remains when we turn from industrial production to the analytical processes. In fields like chemical analysis,^10^ clinical diagnostics,^11^ environmental monitoring,^12^ and forensic testing, the environmental impact is not driven by bulk materials or long-term energy use of a physical product, but by factors such as how samples are collected, transported, stored, and prepared,^13^ which reagents and instruments are used, how waste is disposed, and how the entire workflow is assessed and improved over time. Standard LCA frameworks, designed for tangible goods with well-defined supply chains,^14^ struggle to capture these details because they lack stages that correspond to the unique realities of the analytical laboratory.

Because a customized approach linking LCA principles to the complete analytical workflow is missing, in this work, we introduce the analytical LCA (ALCA). ALCA adapts the core phases of traditional LCA to the analytical context by proposing a coherent sequence of 10 distinct stages that mirror the actual journey of a sample from the field or clinic to the final reported result and beyond. The first stage is sampling,^15^ where a representative portion of a target material is collected from a larger population or environment. The second stage is transportation,^16^ which involves the physical movement of collected samples to the laboratory. The third stage is storage,^17^ where samples are held under controlled conditions before analysis. The fourth stage is pretreatment, which occurs only when the materials needed for sample preparation must themselves be laboratory-prepared or synthesized. The fifth stage is sample preparation,^18^ where the raw sample is extracted, purified, concentrated, or derivatized. The sixth stage is premeasurement, which applies only when a material required for analysis must first be prepared or characterized.^19^ The seventh stage is measurement, where the prepared sample was introduced into an instrument. The eighth stage is waste disposal, which cumulatively handles all of the waste generated across the previous stages. The ninth stage is assessment, where the entire workflow is evaluated across three dimensions: reliability and validation (redness),^20,21^ practicality (blueness),^22,23^ and environmental impact (greenness),^24,25^ integrated through the white analytical chemistry principle.^26,27^ The 10th stage is reporting and decision making, where analytical results are communicated and, more importantly, where the analytical process itself is critically examined to identify opportunities for improvement. By structuring ALCA around these 10 stages, this tool bridged the gap between industrial/managerial LCA and bench-level analytical practice, enabling systematic tracing of environmental burden from sample collection to process improvement.

It is crucial to distinguish ALCA from existing greenness and whiteness assessment tools that have become widely adopted in analytical chemistry. These tools do not indicate which stage in the analytical life cycle needs further improvement or account for most of the environmental burden. ALCA addresses this gap by providing a framework that traces environmental burdens from the moment a sample is collected in the field to the moment decisions are made about process optimization. It is noteworthy that ALCA does not replace these established tools; rather, it incorporates them as complementary modules within its assessment stage (stage 9), where MoGAPI, AGREE, BAGI, RAPI, RGBfast, and other metrics can be applied to evaluate the method while ALCA provides the broader life cycle context.

## Discussion of the 10 Stages of ALCA

### Sampling as the First Stage of ALCA

In the framework of ALCA, sampling is defined as the systematic process of collecting a representative portion of a target material from a larger population, environment, or process stream while preserving its integrity for subsequent laboratory analysis.^28,29^ Unlike traditional LCA, which often begins at raw material extraction for a product, ALCA recognizes that for most analytical workflows, the act of obtaining the sample is the true cradle of the assessment.^30^ Sampling bridges the physical world to the analytical measurement and, if poorly designed, can dominate the total environmental footprint of an analytical process. Several distinct types of sampling exist within ALCA, each carrying its own environmental implications. Discrete or grab sampling involves collecting a single sample at a specific location and time; this approach is simple and requires minimal equipment such as bottles or syringes, but it may demand many individual samples to capture spatial or temporal variability, thereby multiplying sampling events, transport trips, and container use. In contrast, composite sampling combines multiple subsamples from different locations or times into one homogenized sample, which reduces the number of samples sent to the laboratory and, thus, lowers transport and storage impacts. However, composite sampling often necessitates field homogenization equipment, larger containers, and preservatives, creating a trade-off between reduced downstream analysis and increased per-sample field processing. Passive sampling^31^ offers another alternative, where analytes accumulate onto a deployed device over hours to weeks without active pumping, eliminating energy use for pumping during collection but carrying a high upstream environmental cost from the manufacturing of samplers, with long deployment times risking loss or biofouling that may lead to resampling. Finally, active or pumped sampling^32^ uses a device such as an air or water pump to actively draw the matrix through a collection medium, providing controlled volumes and short sampling times but introducing electricity consumption from batteries or generators, noise, and maintenance of pumps, all of which contribute directly to the ALCA inventory.

The way sampling is designed and executed can influence the results of an ALCA in profound ways, often far more than subsequent laboratory analysis. One of the most significant factors is the dominance of transport-related impacts, where the number of sampling locations and the distance between them dictate fuel use, vehicle emissions, and travel times. Ignoring sampling in an ALCA would falsely attribute all environmental impact to the instrument run. Another major consideration is the material footprint from single-use consumables such as gloves, tubing, sterile bags, vials, coolants, and chemical preservatives, which in an ALCA can dominate categories such as abiotic depletion and freshwater ecotoxicity. For example, 100 water samples with presterilized one-liter plastic bottles carry a plastic mass and manufacturing energy equivalent to dozens of analytical injections. Sampling is, therefore, neither a trivial prelude nor an interchangeable step; it is the first and often most environmentally decisive stage, and any ALCA that excludes it risks a fundamental misallocation of the environmental burden. It is worth clarifying that in the ALCA framework, the functional unit is defined per one sample analyzed. This functional unit was selected because it is the most widely used basis for comparing analytical methods and laboratory workflows, enabling consistent quantification of resource consumption, energy use, waste generation, and associated environmental impacts across all stages of the analytical process.

### Transportation as the Second Stage of ALCA

Following the collection of a representative sample, transportation emerges as the second critical stage in the ALCA. Transportation in this context is defined as the physical movement of collected samples from their point of origin to the laboratory where analysis will occur, as well as any intermediate movements between sample preparation, storage, and analytical facilities.^33^ Unlike traditional LCA, where transportation often refers to the shipping of raw materials or finished goods across long supply chains, transportation in ALCA operates at a different scale and with unique constraints, as the sample is typically small in mass and volume yet often perishable, chemically unstable, or biologically active, requiring movement under controlled conditions. Several distinct modes and patterns of transportation appear within the ALCA, each with its own environmental signature. Road transport^34^ using gasoline or diesel vehicles offers flexibility and accessibility but brings fuel consumption, tailpipe emissions, and the infrastructure of packaging and vehicle maintenance. Air transport,^35^ while less common due to its high cost, becomes necessary when samples must travel long distances rapidly, carrying a very high carbon footprint per kilogram per kilometer, often an order of magnitude greater than road transport. Rail and marine transport are rarely used for analytical samples because of their slow speed, but they may appear in very large-scale monitoring programs.^36^ Beyond these conventional modes, ALCA must also account for multimodal transport chains, where a sample might travel by foot, bicycle, car, aircraft, and van in sequence, each segment adding its own emissions and risks.

The way transportation is organized directly affects ALCA outcomes through several interconnected mechanisms. The most obvious is the distance traveled and the associated fuel consumption, though ALCA reveals that this relationship is not linear because the first few kilometers often carry the highest per-kilometer burden due to cold starts, urban congestion, and the overhead of packaging. The choice of vehicle matters enormously, as transporting a single sample in a dedicated car may consume several megajoules of fuel per kilometer, whereas consolidating dozens of samples into one courier trip distributes the impact across many analytical results, introducing a key trade-off between timeliness and efficiency. Another critical factor is the need for temperature control during transit, which can overshadow the propulsion energy itself. Many analytical samples must be maintained at specific temperatures, requiring insulated shipping containers with phase-change materials, gel packs, dry ice, or liquid nitrogen, along with real-time temperature monitoring devices. In ALCA terms, these materials carry their own production and disposal burdens, and a shipment that appears efficient in terms of distance and fuel may have a hidden climate impact from its refrigerants and coolants. Similarly, the packaging materials used to protect samples from breakage all contribute to the material footprint of the transportation stage, with single-use items often discarded after a single journey, adding to resource depletion and litter. Finally, once samples arrive at the laboratory, packaging materials and coolants become waste, and in a comprehensive ALCA, the disposal or reuse of transport consumables must be included as part of the transportation stage’s life cycle, closing the loop from departure to final waste treatment.

### Storage as the Third Stage of ALCA

Once a sample is collected and transported to the laboratory, it rarely moves immediately into the analytical instrument. Instead, it enters the third stage of the ALCA: storage. Storage^37^ is defined as the temporary holding of samples under controlled conditions between their arrival at the laboratory and the moment of analysis, as well as any intermediate storage during sample preparation, extraction, or cleanup steps, including the long-term storage of aliquots, extracts, or reference materials for potential reanalysis or regulatory retention.^38^ Unlike traditional LCA, ALCA must confront the reality that analytical samples are often stored for hours, days, weeks, or even years before measurement, during which time energy is consumed continuously, consumables are deployed, and the risk of sample degradation increases. Several distinct types of storage appear within the ALCA, each with different environmental demands. Refrigerated storage, typically between two and eight degrees Celsius, slows biological activity and chemical degradation but requires constant energy input to run compressor-based cooling units. Frozen storage, ranging from minus 20 degrees Celsius for many environmental and food samples to minus 80 degrees for biological specimens, is extremely energy-intensive, with ultralow temperature freezers often consuming as much electricity per day as a household and generating significant waste heat that must be removed by laboratory air conditioning. Beyond cold storage, some samples require ambient but controlled storage in low-humidity cabinets, opaque containers, or with chemical preservatives, while archived storage keeps extracts or digested residues for years, slowly accumulating an environmental balance that grows with each day of preservation.

The way that storage is managed profoundly affects ALCA outcomes through several interconnected mechanisms. The most direct and often largest impact comes from the continuous energy consumption of refrigeration and freezing equipment, which, unlike transportation, consumes energy relentlessly for as long as the sample remains in the laboratory. A sample stored for 1 week in a minus 80 degree freezer may require more energy for its storage than for its collection, transport, and analysis combined, creating a strong incentive to minimize storage time and to analyze samples as soon as possible after arrival. However, this must be balanced against the efficiency of batching, as running an analytical instrument for a single sample is often energy-inefficient, leading laboratories to accumulate samples until they have enough to justify a batch analysis. ALCA reveals the optimum point where the energy saved by batching outweighs the additional storage energy, a calculation that is unique to each combination of sample type, storage temperature, and analytical method. Beyond energy storage, materials such as plastic vials, cryovials, freezer racks, cardboard boxes, and barcode labels are used, each with its own manufacturing footprint, most of which are single-use and eventually become waste. The chemical preservation of samples adds another layer, as preservatives such as nitric acid or formaldehyde are manufactured through energy-intensive processes, are often toxic, and, after analysis, require hazardous waste disposal, creating a trade-off between chemical toxicity and climate change impacts. Perhaps the most insidious effect is the risk of sample degradation during prolonged holding, where if a sample becomes invalid, the entire analytical process from sampling through transport to storage must be repeated, with the environmental cost, including not only the storage energy consumed but also the complete reinstitution of all previous stages. Storage is, therefore, not a passive waiting period but an active, energy-intensive, and risk-laden stage of the analytical life cycle.

### Pretreatment as a Possible Fourth Stage of ALCA

Not every analytical workflow includes an additional step beyond sampling, transportation, and storage, but when it does, that step must be recognized as a possible fourth stage of ALCA: pretreatment. Pretreatment is defined as the deliberate preparation, synthesis, purification, or conditioning of the materials that will later be used during sample preparation. Unlike sample preparation itself, which involves applying ready-made reagents or sorbents to the sample, pretreatment focuses on creating those very substances from simpler starting materials. This stage is not always present, because many laboratories purchase commercially available reagents, prepacked columns,^39^ or ready-to-use solutions, in which case the environmental burden of producing those items is captured in the inventory of the purchased product. However, when a laboratory synthesizes its own derivatization agents, purifies solvents, grows microbial cultures, fabricates solid-phase extraction sorbents,^40^ prepares buffer solutions from solid salts, or produces ultrapure water internally, then pretreatment becomes an active stage within the ALCA boundary. Several distinct types of pretreatment can appear within ALCA, depending on the analytical method and the laboratory’s degree of self-sufficiency. Solvent purification involves distilling or filtering commercially available solvents to remove impurities, consuming energy for heating, cooling, and pumping, and generating hazardous distillation residues. The synthesis of derivatization reagents combines precursor chemicals under heating and stirring, followed by purification, adding energy consumption, material inputs, and waste streams. The preparation of solid-phase extraction sorbents by functionalizing silica gel or polymerizing monomers can be energy-intensive and involve toxic reagents. The production of ultrapure water using reverse osmosis, electrodeionization, and ultraviolet irradiation consumes electricity continuously, produces wastewater, and requires periodic replacement of cartridges and lamps. The growth and maintenance of biological cultures or cell lines for assays requires incubators, shakers, and sterilized media, consuming energy and materials over days or weeks before any actual sample analysis begins.

The way pretreatment is performed can affect ALCA outcomes through several important mechanisms. The most obvious is the addition of energy consumption that would otherwise be absent, as a laboratory that distills its own solvents uses a heating mantle, cooling water, and a fraction collection system for hours per batch, whereas purchasing predistilled solvents shifts that energy burden to the chemical manufacturer. Whether this shift is environmentally beneficial depends on economies of scale, as large chemical manufacturers often operate more energy-efficient distillation columns, but they also add transport emissions. Another critical factor is the yield and efficiency of pretreatment reactions, where a low-yield synthesis has a much higher environmental burden per gram of useable reagent because more input materials are required to produce the same amount of final product, a burden that must then be allocated across the analytical samples that eventually use that reagent. A subtle but important consideration is that pretreatment can sometimes be avoided entirely through careful method selection and purchasing choices, and from an ALCA perspective, the most sustainable option is not always to perform pretreatment efficiently but to question whether pretreatment is needed at all.

### Sample Preparation as the Fifth Stage of ALCA

Following any necessary pretreatment of reagents and materials, the analytical workflow moves to what is often the most resource-intensive and chemically diverse stage of ALCA: sample preparation, also termed sample treatment. Sample preparation^41,42^ is defined as the set of operations performed on the raw sample after storage and before instrumental analysis, with the purpose of extracting, isolating, purifying, concentrating, or chemically modifying the target analytes so that they are compatible with the chosen measurement technique. This stage transforms a complex, often heterogeneous matrix into a clean, homogeneous, and instrument-ready solution.^43^ At this stage, the vast majority of solvents, reagents, solid-phase materials, and disposable labware are consumed, and the largest volumes of hazardous waste are generated.^44^ Yet, it is also the stage that can be completely avoided in certain fortunate cases, a possibility that ALCA highlights as a powerful lever for sustainability.^45^ The types of sample preparation include liquid–liquid extraction, which consumes large volumes of organic solvents in multiple repeated extractions, solid-phase extraction, which greatly reduces solvent consumption but introduces disposable plastic cartridges and vacuum manifolds, pressurized fluid extraction, which uses elevated temperature and pressure to reduce solvent volumes and extraction times but consumes electricity for heating and pumping, microwave-assisted extraction,^46^ which reduces solvent use at the cost of higher electricity demand, dispersive solid-phase extraction,^47^ which is fast and uses minimal solvent but generates solid waste, derivatization, which consumes additional, often toxic, reagents, and simple dilution or filtration, which consumes solvent and filter membranes.

The way that sample preparation is designed and executed can affect ALCA outcomes more profoundly than almost any other stage. The most significant factor is the solvent consumption and its associated waste. In ALCA, switching from liquid–liquid extraction to solid-phase extraction can reduce the solvent-related global warming potential by a factor of 10 or more. Another major factor is the generation of solid waste from disposable consumables such as pipet tips, syringes, filters, vials, cartridges, and centrifuge tubes, each manufactured from plastic resins, molded using electricity, and after a single use incinerated or landfilled. The energy consumption of sample preparation is often overlooked but can be significant, with techniques such as pressurized fluid extraction, microwave-assisted extraction, and rotary evaporation using electricity for heating, cooling, and mechanical motion, all multiplied by the number of samples and the carbon intensity of the local electricity grid. Perhaps the most important insight that ALCA brings to sample preparation is the recognition that this stage can sometimes be evaded altogether through techniques that allow the direct injection of the raw sample into the instrument without any prior treatment. Headspace gas chromatography, purge-and-trap gas chromatography, ambient ionization mass spectrometry, laser ablation inductively coupled plasma mass spectrometry, and direct injection liquid chromatography all eliminate or dramatically reduce the level of sample preparation. From an ALCA perspective, avoiding sample preparation entirely is usually environmentally beneficial, eliminating not only the direct burdens of solvents, consumables, energy, and waste but also the indirect burdens of pretreatment if that stage was present. However, direct injection methods sometimes suffer from matrix effects that can lead to inaccurate results and reanalysis, and some require specialized instrumentation with a higher manufacturing footprint.

### Premeasurement as a Possible Sixth Stage of ALCA

After sample preparation has rendered the extract or solution ready for instrumental analysis, one might assume that the next logical step is simply to inject the prepared sample into the measuring device. However, there exists a possible sixth stage of ALCA that is often overlooked entirely: premeasurement. Premeasurement is defined as the analytical operations performed on a material that is itself required as an input for the subsequent measurement of target analytes in actual samples. In other words, premeasurement occurs when something that will be used during analysis must first be prepared, characterized, or verified. This stage is not always present, and it enters the ALCA boundary only if the material in question will be prepared before being used in the analytical workflow. For example, if carbon quantum dots are prepared to determine the concentration of an analyte, the synthesis of these carbon quantum dots is considered a premeasurement stage. This should be differentiated from sample preparation in which the sample itself is derivatized, modified, or concentrated, while we herein prepare another material or chemical that aids in the measurement stage. The conditional nature of premeasurement means that ALCA practitioners must carefully examine whether this stage is actually present, asking whether any material that will be consumed or used during the analysis of real samples must first be prepared and characterized to determine its properties. Premeasurement can sometimes be avoided through careful method and material selection. It is important to notice that the pretreatment (stage 4) and premeasurement (stage 6) stages are designated as optional because their applicability depends on the nature of the analytical workflow and the materials employed. When laboratories use ready-to-use products, such as prepacked chromatographic columns, certified reference materials, or commercially prepared reagents, the corresponding preparation activities are performed by the manufacturer rather than the laboratory, which obviates the need to be incorporated in ALCA.

### Measurement as the Seventh Stage of ALCA

After all previous stages have delivered a prepared, and sometimes premeasured, sample to the doorstep of the instrument, the analytical workflow reaches its most recognizable and often most technologically sophisticated stage: measurement, also simply called analysis. Measurement is defined as the instrumental or chemical process by which the presence, concentration, or property of target analytes in the prepared sample is quantitatively or qualitatively determined. This stage transforms a chemically processed extract into a raw signal that is then interpreted into an analytical result. Measurement is the central enabling stage of the analytical workflow, yet it carries its own substantial environmental burden through electricity consumption, carrier and fuel gases, instrument manufacturing and maintenance, and the consumables directly associated with the analytical run. The types of measurement in ALCA are diverse, including chromatographic techniques such as gas chromatography and liquid chromatography, which consume electricity for pumps, temperature control, and detectors, as well as carrier gases like helium, hydrogen, or nitrogen; mass spectrometers, which require high vacuum maintained by continuously running pumps and consumables, such as electron multiplier detectors; optical spectroscopy techniques, including atomic absorption and inductively coupled plasma optical emission, which consume electricity for light sources and, in the case of plasma instruments, large volumes of argon gas; electrochemical techniques, which consume electricity for potential control and often require disposable electrodes; and molecular biology techniques, such as polymerase chain reaction, which consume electricity for thermal cycling and fluorescence detection along with consumables like plastic consumables, enzymes, and nucleotides.

Although calibration and quality control are integral parts of the measurement stage that add additional burden, these steps are not included in ALCA as they should be performed once, while ALCA considers the procedures for a single sample. Moreover, the measurement stage generates waste streams, including mobile phases, rinsing solvents, and detector exhausts, which contribute to impact categories such as human toxicity and ecotoxicity. Measurement is, therefore, far from a purely intellectual activity; it is a physical process with material, energy, and waste consequences that must be honestly accounted for in any comprehensive ALCA.

### Waste Disposal as the Eighth Stage of ALCA

After the measurement is complete and the analytical result has been reported, the journey of the sample and its associated materials does not simply end. The eighth stage of ALCA is waste disposal, defined as the collection, treatment, and ultimate fate of all solid, liquid, and gaseous waste materials generated throughout the entire analytical life cycle. Unlike previous stages that follow a linear sequence, waste disposal stands out as a cumulative stage. Waste is not generated solely at the end of the workflow; rather, it accumulates continuously from sampling, transportation, storage, pretreatment, sample preparation, premeasurement, and measurement itself. Each of these earlier stages contributes its own waste stream, and ALCA recommends that the burdens of handling, treating, and disposing of all of these wastes be aggregated into this single, unified stage. The types of waste encountered in ALCA are remarkably diverse, including solid waste such as empty sample containers, used disposable gloves, packaging materials, freezer racks, used pipet tips, solid-phase extraction cartridges, filter membranes, spent chromatography columns, and worn instrument components; liquid waste such as residual sample material, used solvents, acid digests, buffer solutions, cooling water, and spent chemical preservatives; and gaseous waste, including volatile organic compounds released during sample preparation, carrier gases and detector exhausts, and evaporated solvents.

The cumulative nature of waste disposal in ALCA means that decisions made in earlier stages can have synergistic effects on the disposal burden, as a method using a single recyclable solvent may generate less disposal impact per sample than one using a small volume of a mixed, nonrecyclable solvent mixture. By aggregating all waste disposal efforts into this cumulative stage, ALCA encourages laboratories to think holistically about the full life cycle of their materials, from the moment a sample is taken to the moment its remnants are finally transformed into emissions, ash, or recycled products.

### Assessment as the Ninth Stage of ALCA

After waste disposal has closed the physical loop of the analytical life cycle, one final stage remains, and it is fundamentally different from all of those preceding it. Method assessment is defined as the systematic, multidimensional evaluation of the entire analytical workflow, integrating not only environmental impact but also analytical performance and practical feasibility. Unlike the previous eight stages, which involve the physical handling of samples, materials, and wastes, assessment is an intellectual and comparative activity that asks whether the analytical method is fit for its intended purpose, in the broadest sense. Within ALCA, three main aspects are considered: The first aspect is reliability and validation,^20,21^ captured under the metaphor of redness, which concerns whether the analytical method produces results that are trustworthy, accurate, precise, and defensible for the intended application, including parameters such as limit of detection, limit of quantification, linear range, selectivity, accuracy, precision, robustness, and measurement uncertainty.^48^ A method that has been properly validated produces results that can be relied upon for decision making, and redness matters because an unreliable method may lead to false negatives or false positives, with consequences that extend beyond the analytical laboratory and may require reanalysis with its multiplied burden. The second aspect is practicality, represented as blueness,^22,23^ which concerns the operational and economic feasibility of the analytical method in a real-world laboratory setting, including factors such as time per sample, ease of training, availability and cost of instruments and consumables, throughput, automation potential, space requirements, safety hazards, and compatibility with existing infrastructure. A method that is extremely green and perfectly reliable may still be impractical if it requires rare and expensive equipment, takes too long, or uses reagents that are not commercially available. The third aspect is environmental impact,^49,50^ or greenness,^42^ which is precisely what the previous eight stages of ALCA have been designed to quantify, encompassing the cumulative burdens from sampling through waste disposal aggregated into impact categories.

The integration of these three aspects is achieved through the white analytical chemistry^51^ principle, which holds that a truly excellent analytical method is not one that maximizes any single attribute but one that achieves an optimal balance among reliability, practicality, and environmental impact. The metaphor of white light is deliberate, as white light contains all colors of the spectrum in balance, just as a white analytical method contains redness, blueness, and greenness in proportions appropriate for the analytical task. A method that is extremely green but fails validation is not white; it is simply green and useless. A method that is highly reliable but so impractical that it cannot be completed in time is not white. A method that is very practical but produces unreliable results is not white. The white method is one where all three aspects have been considered and optimized together, recognizing that the optimal balance depends on the specific analytical question. Within the assessment stage of ALCA, the white analytical chemistry principle provides a decision framework. For a given analytical problem, one might define minimum acceptable levels of redness and blueness and then seek the method that achieves these with the lowest possible greenness impact, or alternatively balance trade-offs when no single method dominates. The assessment stage also includes comparative evaluation across multiple candidate methods, potentially revealing that the method with the lowest greenness impact is not chosen because its redness is marginal, or its blueness is poor.

Within the assessment stage, several established metrics can be employed to quantify the three dimensions of whiteness. Among these, the RGBfast model, developed by Nowak and Arduini, offers a user-friendly and automated approach. RGBfast evaluates analytical methods across six key criteria: three red criteria (trueness, precision, and limit of detection), one green criterion (the ChlorTox scale, which integrates reagent hazards and quantities), and two blue criteria (sample throughput and energy consumption). The model generates a comprehensive “whiteness” score that enables simultaneous consideration of analytical quality, environmental sustainability, and practical feasibility. Its automated Excel-based implementation eliminates subjective point awarding and provides concise, interpretable pictograms. Within the ALCA framework, RGBfast can serve as a complementary module during the assessment stage, providing a standardized and rapid evaluation of the method’s whiteness that integrates seamlessly with the broader life cycle perspective offered by ALCA. The RGBfast results, together with other metrics such as MoGAPI, AGSA, AGREE, BAGI, CACI, AMRI and RAPI, contribute to a comprehensive, multifaceted assessment of the analytical workflow, ensuring that sustainability improvements are pursued without compromising analytical reliability or operational feasibility.

It is worth clarifying that, whenever possible, ALCA should be populated using primary, laboratory-specific data to maximize the accuracy and representativeness of the assessment. However, when such data are unavailable or impractical to obtain, validated literature values, supplier information, or well-justified default values may be used, provided that the data sources and underlying assumptions are clearly documented. As higher-quality primary data become available, the assessment can be refined to improve the reliability of the results while maintaining the practical applicability of the ALCA framework.

### Reporting/Improving and Decision Making as the 10th Stage of ALCA

After assessment has integrated the three pillars of redness, blueness, and greenness into a white analytical evaluation, the analytical life cycle arrives at its final and arguably most consequential stage: reporting and decision making. Reporting and decision making are defined as the formal communication of analytical results to stakeholders, combined with the systematic evaluation of the entire analytical process itself to identify opportunities for improvement, optimization, or redesign. The first purpose is the conventional one: reporting the concentration, presence, or property of target analytes in the original samples, along with measures of uncertainty, so that informed decisions can be made about product quality, environmental compliance, patient health, or food safety. The second purpose, unique to ALCA and often overlooked in routine analytical practice, is the meta decision about the analytical process itself. On the basis of the quantitative and qualitative information gathered across the previous nine stages, the analyst or laboratory manager asks transformative questions. Can the analytical process be improved? Which stages are the most energy-intensive, material-consuming, or waste-generating? Can these stages be evaded entirely or, if not, can they be minimized? Is there a whiter alternative that balances reliability, practicality, and environmental impact better than does the current method? The reporting and decision-making stage thus closes the loop, turning ALCA from a descriptive exercise to a prescriptive and continuous improvement tool.

Beyond decisions about individual methods, the reporting and decision-making stage can drive systemic changes in laboratory culture and infrastructure such as implementing automatic instrument shutdown protocols or installing solvent distillation units. By embedding reporting and decision making as an integral stage, ALCA ensures that each analytical campaign contributes not only to knowledge about the sample but also to knowledge about how to make the next campaign more sustainable.

It is essential to demarcate clearly the distinct roles of stage 9 (assessment) and stage 10 (reporting and decision-making) within the ALCA framework, as they serve complementary but fundamentally different purposes. Stage 9 provides the “what” and “how much” (quantitative assessment), while stage 10 provides the “so what” and “now what” (actionable decisions and future planning). This distinction is crucial for ensuring that ALCA is not merely an academic exercise but also a practical, iterative tool for continuous improvement, where each cycle of assessment informs the next round of decision-making and process revision.

## Case Studies: Applying ALCA with the Dual-Threshold Rubric

To illustrate how the 10-stage ALCA framework functions in practice, five hypothetical case studies are presented below. Each case follows the same analytical scenario: a routine environmental monitoring laboratory that measures pesticide residues in surface water samples collected from 10 remote river locations, processing approximately 500 samples per year. However, each case applies ALCA with a different focus on energy consumption, water consumption, carbon footprint, active analyst time, and cost. For each case, the assessment is performed using the dual-threshold rubric. This rubric has two components. The first component is relative: the average contribution per stage is calculated as the total divided by the number of stages present. Any stage contributing more than twice the average is considered a relative hotspot. Any stage contributing less than half the average is considered a relative low contributor. The second component is absolute: based on benchmarking against best available practices, industry standards, or science-based targets, each metric has absolute green, yellow, and red zones. A stage is classified as red if it exceeds the absolute red threshold or if it is a relative hotspot with an absolute value that is not already green. A stage is classified as yellow if it falls between absolute thresholds or is moderately above average without reaching the red zone. A stage is classified as green only if it meets absolute best practice and is not a relative hotspot. The total process is also classified based on the sum of all stages against the absolute benchmarks. It is worth clarifying that the selection of the relative hotspot threshold at twice the average stage contribution is a deliberate and logic-based decision, rooted in both practical analytical experience and established principles of process optimization. This threshold, which flags any stage contributing more than 2× the mean per-stage impact, is set to identify outliers that are disproportionately large compared to other stages. The choice of a 2× multiplier, rather than a more stringent value like 1.5× or a more permissive one like 3×, strikes an essential balance between sensitivity and applicability. A lower threshold would result in an excessive number of “hotspots” being flagged, overwhelming laboratories with a diffuse set of improvement targets and potentially diluting focus on the most critical issues. Conversely, a higher threshold could permit a truly dominant stage to remain concealed within a sea of smaller contributions, delaying meaningful intervention. Furthermore, this relative approach is inherently adaptive; as a laboratory makes improvements and reduces the overall impact, the average decreases, and the 2× threshold recalibrates dynamically. This prevents a stage from being permanently classified as a “hotspot” following a single improvement, thereby promoting continuous improvement rather than isolated corrective actions. For cases 1–4, the assessment is based primarily on the relative average method (hotspot identification), while absolute benchmarks are used only for the total process classification. For case 5 (cost), an absolute benchmark is applied to show how ALCA can be adapted according to the intended purpose.

### Case Study 1: Assessment of Energy Consumption

Turning first to energy consumption, the laboratory begins by collecting data across all 10 stages. Sampling consumes 15.2 kWh per sample, transportation adds 2.8 kWh, storage contributes 2.25 kWh, pretreatment adds only 0.042 kWh, sample preparation uses 1.0 kWh, measurement requires 3.0 kWh, waste disposal adds 0.6 kWh, and reporting accounts for 0.07 kWh. Summing these values gives 24.96 kWh per sample. With the average per stage at 2.49 kWh, twice that value is 4.98 kWh, meaning that any stage exceeding this threshold is flagged as a relative red hotspot. Sampling clearly falls into this category, at 15.2 kWh. Measurement and transportation are in the yellow zone, while sample preparation and waste disposal are green. The total of 24.96 kWh falls within the target range, making the overall process yellow. Figure 1 presents the ALCA pictogram for this case, highlighting sampling as the dominant energy hot spot.

**Figure 1:**
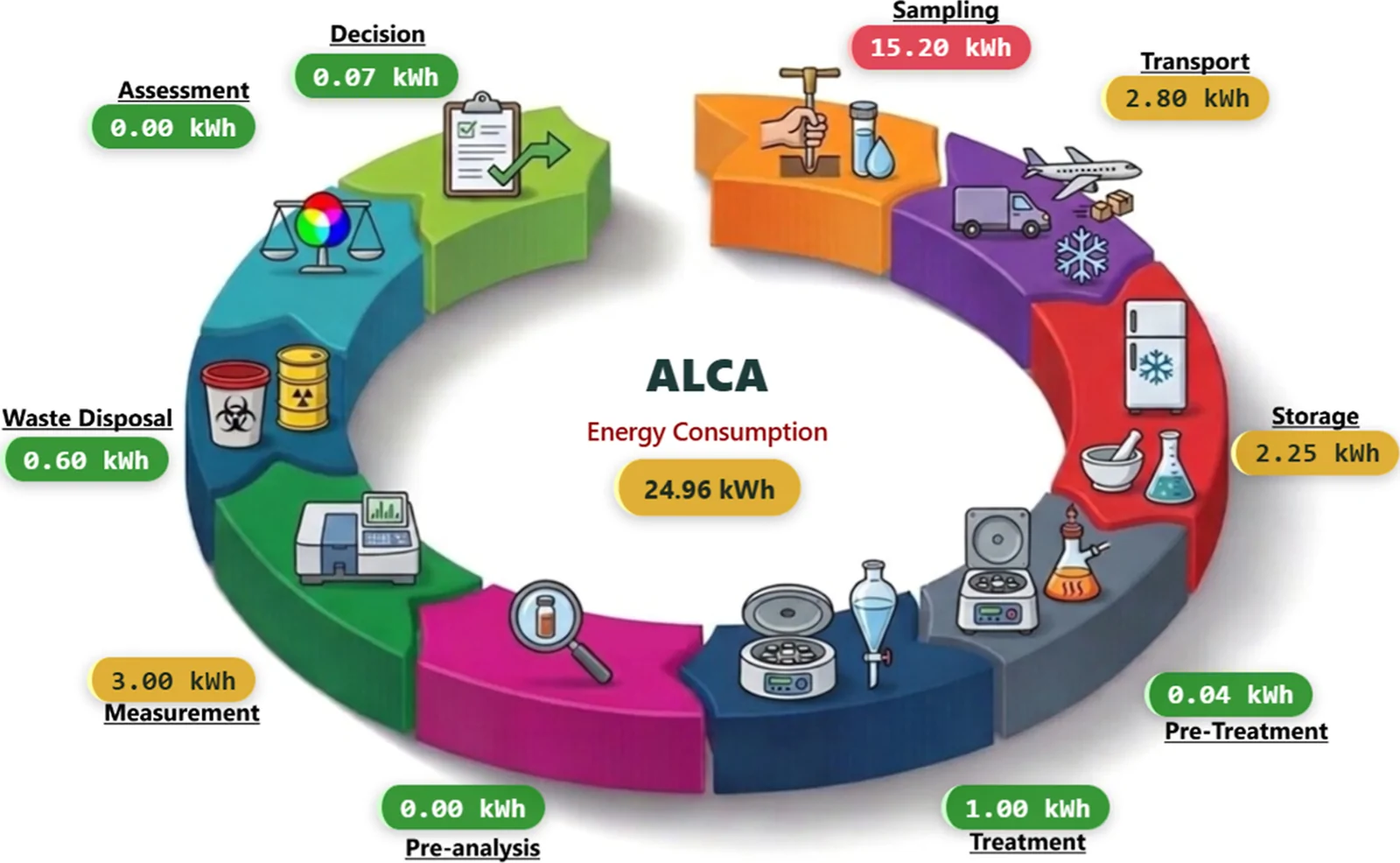
ALCA pictogram for energy consumption showing sampling as the initial red hotspot (15.2 kWh). After trip consolidation and electric vehicle use, the overall process improved from yellow to green.

The target total of 15 kWh was determined based on the laboratory’s historical energy consumption data and the specific instrument requirements for this analytical method. The target accounts for the energy demands of the gas chromatograph–mass spectrometer, the complexity of environmental sample collection requiring multiple field trips, and the refrigeration needs for sample storage. The laboratory first tackled the sampling stage by consolidating field trips, reducing its energy demand to 6.1 kWh. The total dropped to 15.86 kWh, which remained above the 15 kWh target but below double the target (30 kWh), moving the overall process from red to yellow. The new average of 1.59 kWh made twice the average 3.18 kWh, so sampling remained a relative red hotspot despite the improved total. Next, the measurement stage was optimized down to 1.8 kWh. The total became 14.66 kWh, now falling below the 15 kWh target and turning the overall process green. However, with a new average of 1.47 kWh and a doubled threshold of 2.94 kWh, sampling at 6.1 kWh stubbornly stayed in the red. Finally, the laboratory switched to electric vehicles for sample collection, cutting sampling further to 3.2 kWh. The average became 1.18 kWh, twice of which is 2.36 kWh, and sampling finally fell below this threshold, turning yellow. All stages were now green or yellow, and the final energy consumption was 11.76 kWh, well under the 15 kWh target. The laboratory accepted this significant improvement and planned to pursue further reductions in storage and transportation during the next cycle.

### Case Study 2: Assessment of Water Consumption

Shifting focus to water consumption, the laboratory compiles a similar inventory. Sampling uses 2.3 L per sample, transportation adds 0.8 L, storage contributes 0.5 L, pretreatment requires 2.3 L, sample preparation consumes 5.7 L, measurement adds 1.8 L, waste disposal contributes 1.7 L, and reporting adds 0.1 L. The total comes to 15.2 L per sample. With an average of 1.52 L, twice that is 3.04 L; therefore, any stage above this value is a relative red hotspot. Sample preparation (treatment) at 5.7 L clearly qualifies. Pretreatment, sampling, measurement, and waste disposal all fall in the yellow zone, while storage and reporting are green. A total of 15.2 L exceeds double the target, placing the overall process in the red zone. The ALCA pictogram for water consumption is shown in Figure 2, where sample preparation stands out as the primary red stage.

**Figure 2:**
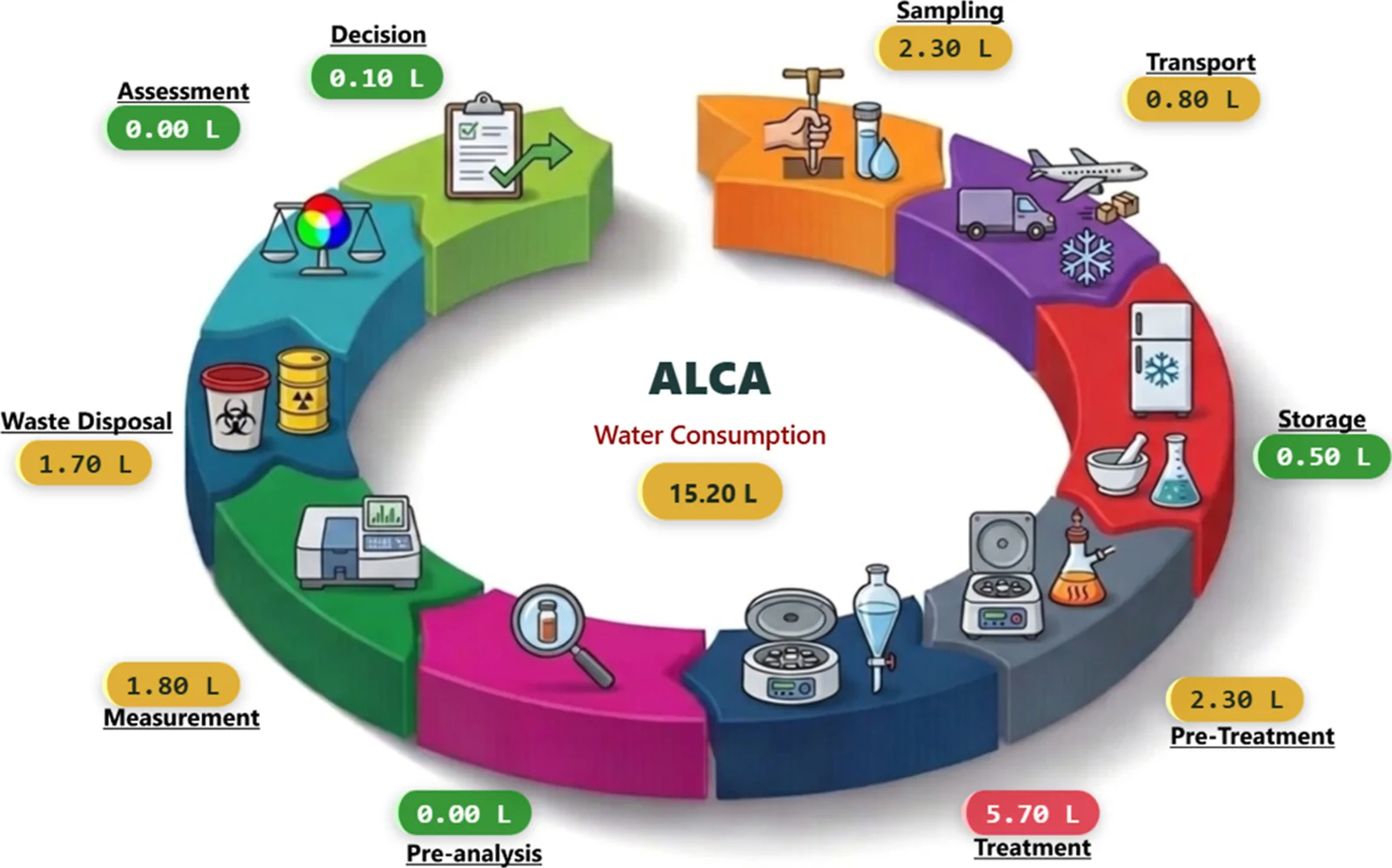
ALCA pictogram for water consumption identifying sample preparation as the red hotspot (5.7 L). Water recycling and bottled ultrapure water reduced the total footprint.

A target total of 10 L per sample was selected as the goal for water consumption reduction. This target was established considering the laboratory’s water recycling capabilities, the availability of bottled ultrapure water alternatives, and the water intensity of the solid-phase extraction method used for sample preparation. The laboratory first targeted sample preparation, introducing a dishwasher that recycles rinse water and cutting its consumption to 3.4 L. The total dropped to 12.9 L, which remained above the 10 L target but below double the target (20 L), placing the overall process in the yellow zone. Recalculating the average gave 1.29 L, with twice that at 2.58 L, so sample preparation at 3.4 L remained above the threshold and stayed a red hotspot. As a proactive next step, the laboratory switched from on-site ultrapure water production to bottled ultrapure water, reducing pretreatment from 2.3 to 1.4 L. The total fell further to 11.0 L, still above the target but within the yellow zone. The new average of 1.10 L set the doubled threshold at 2.20 L, yet sample preparation at 3.4 L persisted as a red hotspot. The laboratory concluded that reaching a total below the 10 L target would require a fundamental method change, such as adopting direct injection techniques that eliminate sample preparation altogether. This option was scheduled for exploration in the next budget cycle.

### Case Study 3: Assessment of Carbon Footprint

For the carbon footprint, the laboratory measures emissions in kg of CO_2_ equivalent per sample. Sampling contributes 4.7 kg, transportation adds 2.1 kg, storage adds 1.1 kg, pretreatment adds just 0.1 kg, sample preparation adds 1.2 kg, measurement adds 1.9 kg, waste disposal adds 0.8 kg, and reporting adds 0.1 kg. The total amount was 12.0 kg. The average per stage is 1.2 kg, so twice the average is 2.4 kg, making any stage above this a relative red hotspot. Sampling at 4.7 kg exceeds this threshold and is, therefore, red. Measurement at 1.9 kg, transportation at 2.1 kg, and sample preparation at 1.2 kg are all yellow or green. The total of 12.0 kg exceeds double the target, so the overall process is red.^52^ Figure 3 illustrates the carbon footprint distribution across the 10 stages, with sampling initially dominating.

**Figure 3:**
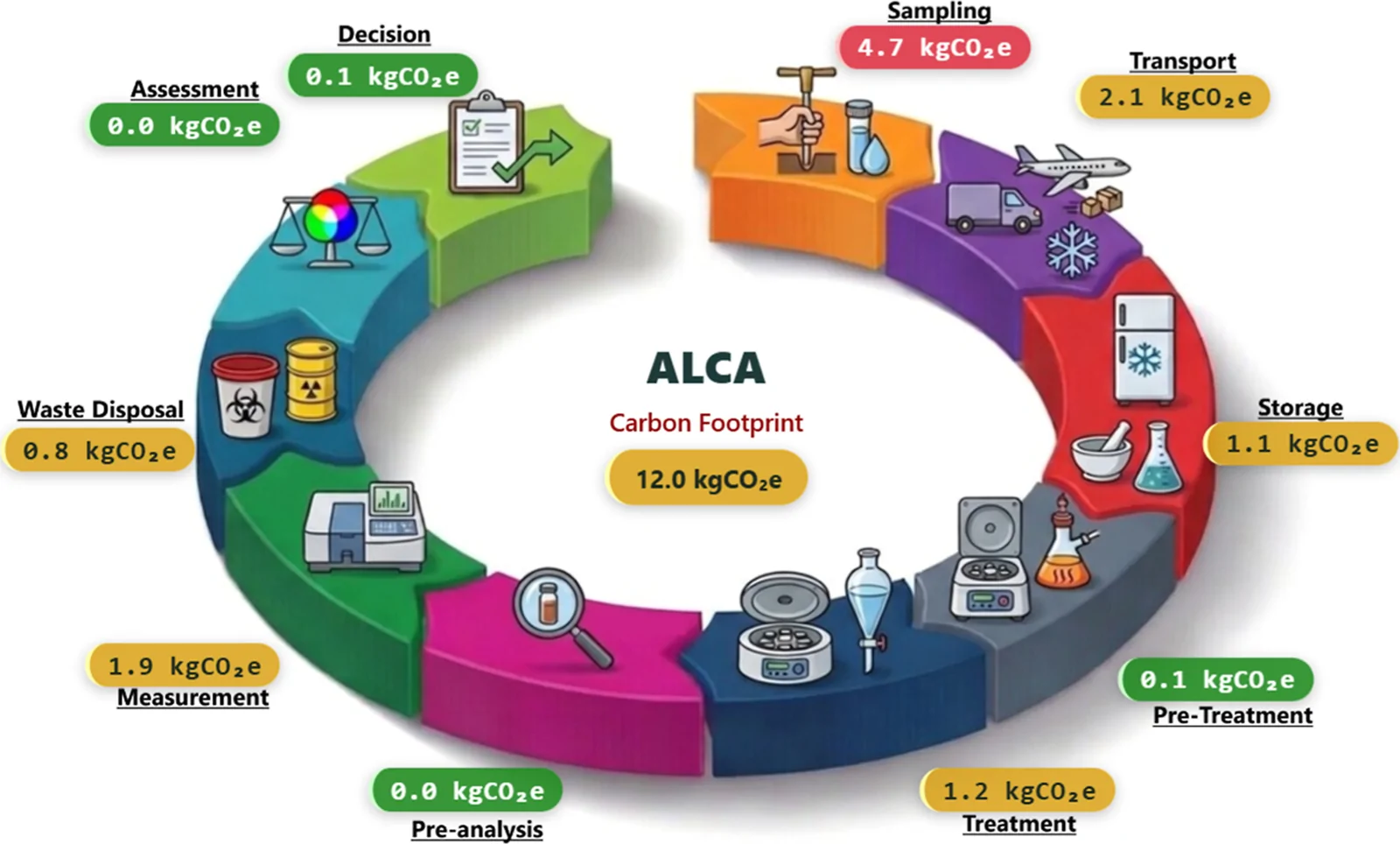
ALCA pictogram for carbon footprint with sampling as the initial red hotspot (4.7 kg CO_2_e). Electric vehicles and hydrogen carrier gas eliminated red stages, though the total remained yellow.

A target total of 8 kg CO_2_e was established as the desired upper limit for the carbon footprint. This target was determined based on the laboratory’s sustainability commitments, the carbon intensity of the local electricity grid, the emissions from the helium carrier gas supply chain, and the travel distances required for sample collection. The laboratory first addressed the sampling hotspot by switching to electric vehicles, reducing this stage to 1.6 kg. The total became 8.9 kg, which was above the 8 kg target but below double the target (16 kg), moving the overall process from red to yellow. The new average of 0.89 kg set the doubled threshold at 1.78 kg, and sampling at 1.6 kg now fell below this value, moving into the yellow zone and losing its red hotspot status. Next, the laboratory replaced helium with hydrogen as the carrier gas in the measurement stage, reducing its footprint from 1.9 to 1.1 kg. The total dropped further to 8.1 kg, still slightly above the 8 kg target but well within the yellow zone. With a new average of 0.81 kg and a doubled threshold of 1.62 kg, all stages were now below the relative hotspot threshold. Nevertheless, the total remained in the yellow zone, and the laboratory recognized that the cumulative effect of many small contributions kept it above the target. Further reductions would require simultaneously addressing transportation and storage in the next improvement cycle to finally achieve the 8 kg goal.

### Case Study 4: Assessment of Active Analyst Time

Moving to active analyst time, the laboratory records the following per sample: sampling takes 48 min, storage requires 2 min, pretreatment takes 0.6 min, sample preparation consumes 28 min, measurement needs 5 min, waste disposal takes 10 min, assessment requires 0.3 min, and reporting takes 5 min. The total active time sums to 98.9 min per sample. The average per stage is 9.89 min, so twice that is 19.78 min, meaning any stage above this is a relative red hotspot. The total exceeds double the target; therefore, the overall process is red. Both sampling at 48 min and sample preparation at 28 min are above the doubled average, making them red hotspots, while waste disposal at 10 min is yellow. The ALCA pictogram for active analyst time is presented in Figure 4, which clearly shows sampling and sample preparation as the two major time burdens.

**Figure 4:**
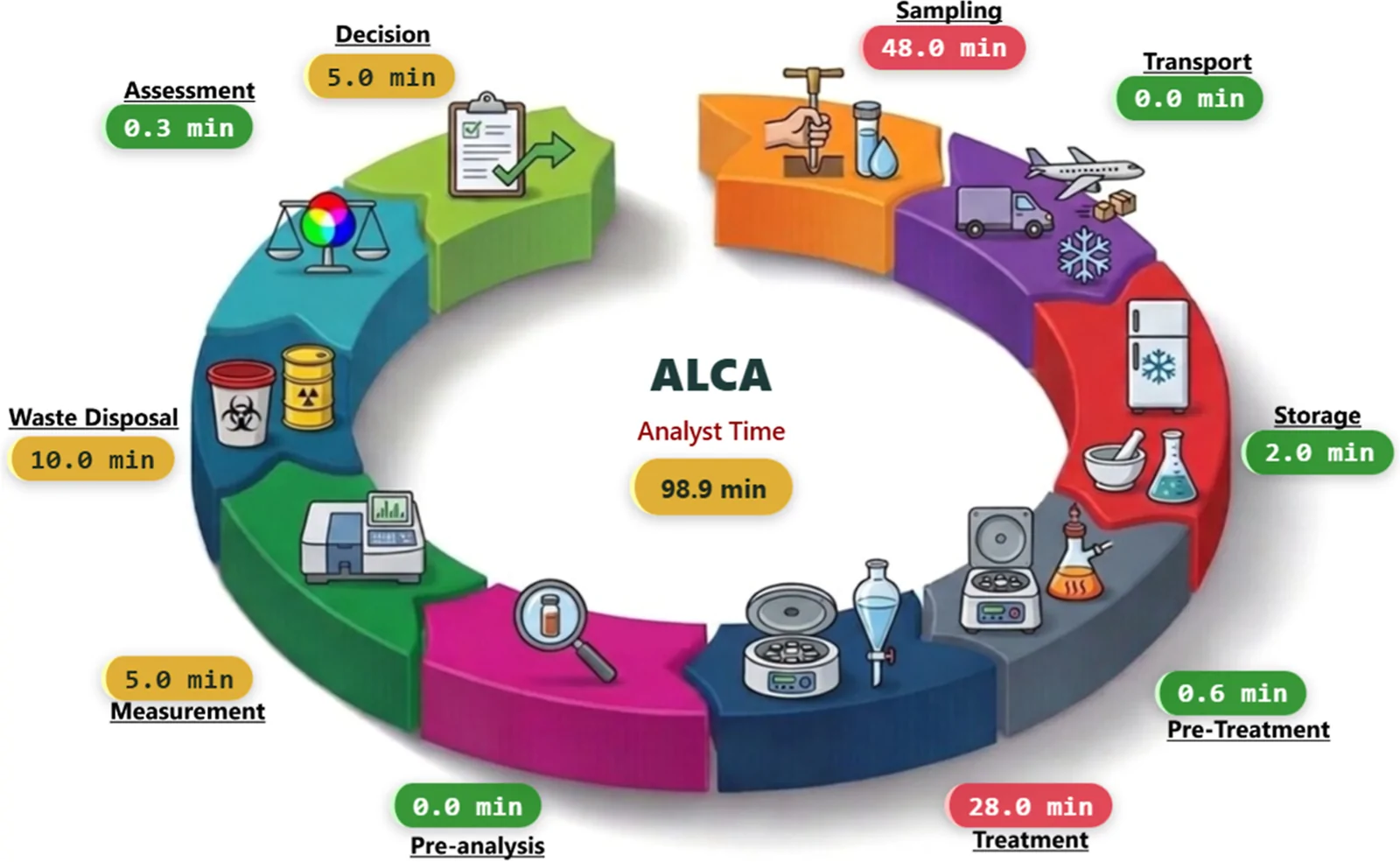
ALCA pictogram for active analyst time showing sampling and sample preparation as red hotspots (48 and 28 min). Automation and trip consolidation left the total in the yellow zone.

A target total of 60 min was set as the maximum acceptable active analyst time. This target was derived from the laboratory’s staffing capacity, the complexity of the analytical procedure requiring multiple manual steps, and the operational need to maintain a reasonable sample throughput of at least 8 samples per 8 h shift. The laboratory first tackled sampling, consolidating trips to reduce its time to 28 min. The total became 78.9 min, which was above the 60 min target but below double the target (120 min), moving the overall process from red to yellow. The new average of 7.89 min gave a doubled threshold of 15.78 min. Sampling at 28 min remained above this and stayed a red hotspot, and sample preparation at 28 min was also above the threshold. Next, the laboratory automated sample preparation, cutting it from 28 to 12 min. The total fell to 62.9 min, which remained just above the 60 min target but still within the yellow zone. The average became 6.29 min, with a doubled threshold of 12.58 min. Sampling at 28 min continued to exceed this value, persisting as the sole remaining red hotspot. Finally, the laboratory implemented a more efficient waste segregation system, reducing waste disposal from 10 to 5 min. The total dropped to 57.9 min, finally falling below the 60 min target and turning the overall process green. Sampling remained above twice the average, but the total was now acceptable. The laboratory concluded that reaching a green total below 60 min would require eliminating storage waiting time entirely through just-in-time analysis. While logistically challenging, this approach was approved for pilot testing on high-priority samples.

### Case Study 5: Assessment of Cost

For the final case, the laboratory assesses the cost using a different approach. Instead of relative averaging, a strict absolute benchmark is applied: less than 1 dollar per sample is green, 1 to 10 dollars is yellow, and more than 10 dollars is red. The sampling stage costs $1.70, placing it in the yellow zone. Storage at 0.50 dollars and pretreatment at 0.32 dollars are both green. Sample preparation at $23.97 exceeds 10 dollars and is therefore red. Measurement at $48.65 is also red. Waste disposal at $8.47 falls in the yellow zone, assessment at $0.30 is green, and reporting at $3.48 is yellow. The total cost sums up to 87.39 dollars per sample. The total exceeds double the target, so the overall process is red. Figure 5 displays the ALCA pictogram for the cost assessment, highlighting how measurement and sample preparation keep the total cost high, despite improvements elsewhere.

**Figure 5:**
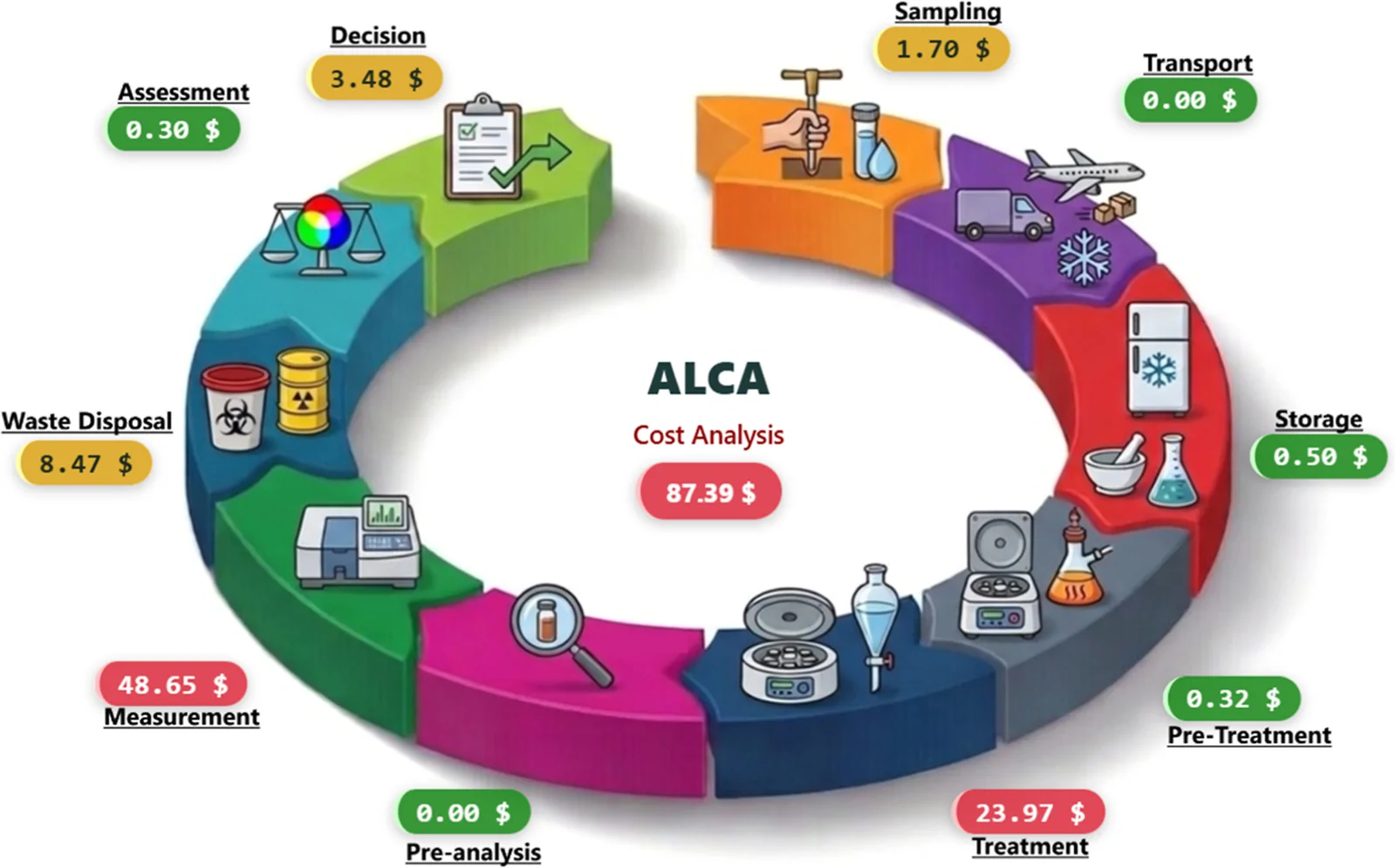
ALCA pictogram for cost using a strict benchmark (red >10 $). Measurement and sample preparation remained red despite reductions, keeping the total process costly.

A target total of 40 dollars per sample was established as the cost reduction goal. This target was determined based on the laboratory’s budget constraints, the capital costs of alternative instrumentation, the availability of less expensive consumables, and the potential for outsourcing certain analytical steps. The laboratory first replaced the gas chromatograph with a less expensive liquid chromatograph, reducing measurement to 22.30 dollars, though this remained above 10 dollars and thus still red. The total became 61.04 dollars, which was above the 40-dollar target but below double the target (80 dollars), moving the overall process from red to yellow. Next, sample preparation was automated, lowering its cost to 10.63 dollars, but this also stayed above 10 dollars and remained red. The total fell to 47.77 dollars, still above the target but within the yellow zone. The laboratory then negotiated lower waste disposal costs, reducing that stage from 8.47 to 4.20 dollars, which moved it from yellow to green. The total became 43.57 dollars, still above the 40-dollar target but in the yellow zone. A further optimization switched the carrier gas from helium to hydrogen, reducing measurement to 20.10 dollars, still red, and bringing the total to 41.23 dollars; still just above the target. At this point, all stages except measurement and sample preparation were green or yellow, yet the total remained above 40 dollars. The laboratory concluded that reaching a green total below 40 dollars was not feasible with current methods. However, achieving a yellow total between 10 and 40 dollars would require a fundamental redesign, such as switching to a direct injection technique that eliminates sample preparation and reduces measurement cost. The laboratory decided to investigate such methods in collaboration with an instrument manufacturer.

The dual-threshold rubric was applied differently across cases. Cases 1 through 4 used the relative average method to identify stage-level hotspots, while case 5 used a revised absolute benchmark for cost. The rubric prevented the problem of all stages appearing green while the total impact remained unacceptably high, because the total process itself was classified against the target totals. The target totals were determined on a case-by-case basis, considering several factors, including the instrument used, the complexity of sampling, transportation requirements, storage conditions, and other operational constraints specific to each analytical method. In the energy case, the total went from yellow to green; in the water case, the total remained yellow; in the carbon case, the total went from red to yellow; in the time case, the total went from red to green; and in the cost case, the total went from red to yellow. Relative hotspot identification guided laboratories to stages that were disproportionately large compared with others. The cost case demonstrated that even when many stages are green or yellow, a few red stages could keep the total above the target under a stringent benchmark. These case studies show that ALCA with the dual-threshold rubric and case-specific target totals is a practical, actionable tool for continuous improvement of analytical sustainability.

To demonstrate the practical applicability of ALCA beyond hypothetical scenarios, the framework was applied to a real analytical method reported in the literature. The menthol-assisted homogeneous liquid–liquid microextraction method was developed as a green and efficient sample preparation technique for the determination of favipiravir in human plasma using HPLC/UV.^53^ The method introduced menthol as a novel phase-separating agent, replacing hazardous chlorinated solvents while enhancing extraction efficiency through reverse micelle formation. Under optimized conditions (300 μL tetrahydrofuran, 30 mg menthol, and 1 min vortexing), the method demonstrated excellent analytical performance, with a linear range of 0.1–100 μg mL^–1^ (*R* ^2^ = 0.9992), recoveries of 97.1–103.9%, and precision below 8.2% RSD. The developed procedure was successfully validated according to FDA bioanalytical guidelines and applied to a human bioequivalence study, providing a simple, sensitive, economical, and environmentally friendly alternative to conventional extraction techniques. From analytical life-cycle assessment, the total water consumption of the analytical workflow was 13.9 mL, with the majority attributed to the pretreatment stage (10 mL), followed by the measurement stage (3 mL) and sample preparation (0.9 mL). No measurable water consumption was associated with sampling, transportation, storage, premeasurement, waste disposal, assessment, or reporting, highlighting that the environmental water footprint of the method is primarily concentrated in laboratory pretreatment operations rather than the extraction process itself.

A target total of 10 mL could be established as the desired upper limit for water consumption, based on the laboratory’s water recycling capabilities and the availability of alternative pretreatment methods. The total consumption of 13.9 mL would exceed this target but remain below double the target (20 mL), placing the overall process in the yellow zone. The pretreatment stage at 10 mL would exceed twice the average threshold of 2.78 mL, making it a relative red hotspot. The measurement stage at 3 mL and sample preparation at 0.9 mL would fall within the yellow and green zones, respectively. To address the pretreatment hotspot, the laboratory could investigate the possibility of recycling wash water through a filtration system, potentially reducing its consumption to 5 mL. This would lower the total to 8.9 mL, bringing the overall process below the 10 mL target and turning it green. The new average of 1.78 mL would set the doubled threshold at 3.56 mL, and pretreatment at 5 mL would remain above this value, persisting as a relative red hotspot despite the improved total. The measurement stage could also be optimized by reducing the mobile phase flow rate from 1.0 to 0.8 mL/min, cutting its water consumption from 3 to 2.4 mL. This would bring the total to 8.3 mL, still below the target but with pretreatment at 5 mL remaining above the doubled average threshold. The laboratory could also explore changing the mobile phase composition to a higher organic solvent ratio, which would decrease the aqueous buffer requirement and reduce pretreatment water consumption. Additionally, implementing a just-in-time mobile phase preparation strategy could eliminate waste from unused buffer solutions. These modifications would be scheduled for investigation in the next method development cycle.

The comparison across all six case studies demonstrates that ALCA with the dual-threshold rubric and case-specific target totals is a versatile, practical tool applicable to both hypothetical assessments and real analytical methods, enabling laboratories to identify environmental hotspots and prioritize improvement strategies for sustainable analytical chemistry. A critical consideration in the practical application of ALCA is the inherent challenge of acquiring accurate, stage-specific data for each of the 10 analytical stages. The framework is designed to be filled with primary, laboratory-specific data to maximize accuracy; however, obtaining such detailed information for every stage can be a significant hurdle. For instance, quantifying the exact energy consumption of a specific freezer over a sample’s storage period, or the precise fuel consumption for a multisite sampling trip, often requires dedicated submetering or meticulous logging that is not standard practice in many laboratories. Similarly, tracking the exact mass of all consumables from pipet tips to SPE cartridges and the precise volume of every solvent used across different procedures can be labor-intensive. For stages identified as major potential contributors, a more targeted effort to collect primary data is important, such as installing a submeter on an energy-intensive instrument, consulting an expert, or implementing a simple waste logging system over a defined period. Stages that generate negligible values of the parameter being evaluated can be excluded from detailed data collection. In this way, ALCA aims to present best practices in reporting, enable comparison between analytical processes and stages, and optimize the whole process based on the collected data. Furthermore, laboratories can adopt best practices to facilitate data collection, ensuring that ALCA remains both an accurate and an actionable tool for continuous improvement.

## Conclusion

ALCA provides a comprehensive stage-by-stage framework for evaluating and improving the sustainability of analytical workflows. The 10 stages, including sampling, transportation, storage, pretreatment, sample preparation, premeasurement, measurement, waste disposal, assessment, and reporting/decision making, capture the unique characteristics of analytical processes that traditional LCA overlooks. By defining each stage, describing its types, and analyzing its potential impacts, ALCA enables laboratories to trace environmental burdens from the moment a sample is collected to the moment decisions are made about process improvement. A critical consideration is the scope boundary of ALCA with respect to method development activities, including optimization trials, unsuccessful experiments, and solvent waste generated during the initial development phase. As these activities are typically performed only once during method development, they should be excluded from ALCA evaluation. Instead, the assessment should focus exclusively on the repetitive procedures carried out during routine analytical applications. The integration of redness (reliability and validation), blueness (practicality), and greenness (environmental impact) through the white analytical chemistry principle ensures that sustainability is not pursued at the expense of analytical quality or operational feasibility. It is worth clarifying that ALCA is intended to complement rather than replace existing greenness, whiteness, and sustainability assessment tools. Within the assessment stage, established metrics such as MoGAPI, AGSA, AGREE, CACI, BAGI, AMRI, RAPI, RGB12, and RGBfast, and related approaches can be incorporated as complementary modules to evaluate the sustainability performance of the analytical method, while ALCA provides a higher-level life cycle framework that integrates these evaluations with environmental impacts arising from the entire analytical workflow. The dual-threshold assessment rubric, combining absolute benchmarks with relative hotspot identification, overcomes the limitations of simple quartile-based methods and provides a clear color-coded pathway for prioritizing improvements. The five hypothetical case studies demonstrate the application of ALCA to energy, water, carbon, time, and cost, showing how the framework identifies hot spots, guides decisions, and drives measurable improvements. ALCA is not a one-time exercise but a cycle of continuous improvement, where each complete pass through the 10 stages yields a more sustainable, more reliable, and more practical analytical process than the last. By adopting ALCA, laboratories can transform their environmental awareness into action, contributing to a more sustainable future for analytical science and the communities it serves.

## Data Availability

No data sets were generated or analyzed during the current study.
